# Language matters: the case of “septic arthritis”

**DOI:** 10.1007/s15010-025-02680-z

**Published:** 2025-11-18

**Authors:** Gerd Fätkenheuer

**Affiliations:** 1 Division of Infectious Diseases, Department I of Internal Medicine, University of Cologne, Faculty of Medicine and University Hospital of Cologne, 50924, Cologne, Germany; 2 Center for Infectious Diseases, University Hospital of Cologne, Cologne, Germany

**Keywords:** Infection, Infectious, Sepsis, Septic, Arthritis, Language

As specialists of Infectious Diseases, we don´t have fancy techniques or apparatus at hand that help us in our diagnostic and therapeutic efforts. Our tools consist of our eyes, our ears, our tongue, our nose, our hands, and of course, our brain. That´s it! As interdisciplinary specialists, we usually share our findings and thoughts with other colleagues. Our advice will only be convincing for them if we argue clearly and precisely. Language matters.

It sometimes begins with a few words. Have you ever been called to treat a patient with the diagnosis of “septic arthritis”? I guess you have. Eventually, you are told that *Staphylococcus aureus* has grown from pus from a knee puncture. In other cases you will be informed that the purulent material aspirated during arthroscopy remained sterile. The diagnoses you are confronted with are the same: “septic arthritis”. I think we can immediately agree upon “arthritis”. But “septic”? You didn´t meet the patient in a condition you would rate as “sepsis”. They were awake, in a good mood, and without any abnormality of their vital signs.

As a physician with many years of clinical practice, I have experienced several definitions of “sepsis” over time. When I started my training in Internal Medicine in the 1980s, “sepsis” was not clearly defined. Instead, it was mainly a synonym of bloodstream infections, and the term “septicaemia” (which literally means “sepsis in the blood”!) was also used for bacteraemia. The usage of the adjective “septic” was even broader. “Septic delivery”, “septic thrombosis”, “septic arthritis” or the more general “septic condition” were used for disorders that did all not necessarily fulfil our current definition of sepsis, but did just indicate an infectious disease. “Septic” in combination with “abscess” was used to suppose a “metastatic” (another very questionable word in this context) infection as a sequel for bloodstream infection. Even theatres could be “septic”! As for describing a patient in a clinically unstable status, colleagues often called him or her as “pre-septic” during handover.

As long as we had no general definition and no deeper understanding of sepsis as its own condition, this wild and vague language may have been acceptable. But things are different nowadays.

Now, with the “Third International Consensus Definitions for Sepsis and Septic Shock” released in 2016, we have a clear definition of sepsis that should also steer the use of this word and of all terms derived from it: “Sepsis should be defined as life-threatening organ dysfunction caused by a dysregulated host response to infection.” [1] But we are far away from a stringent use of “sepsis” in our language, and the reported experience on the ward is not at all unique. If you search PubMed for “septic arthritis”, after 2016 you get 8,766 hits [2], and you will also find 964 papers listed for “septic thrombosis” [2]. Even in journals with a focus of infectious diseases, papers with “septic” conditions in the title that describe entities clearly beyond the definition of sepsis have recently been published [3–8].

Whether or not a patient has sepsis makes a big difference in the management of infectious diseases – in arthritis as well as in other infections. Therefore, we should differentiate these conditions thoroughly when speaking of them. Language reflects our thinking, and our thinking steers our actions. Thus, insisting on clear and precise language is not only an academic exercise. In medicine, and especially in the field of infectious disease, it can be crucial for the outcome of a patient. As specialists in infectious diseases, we should use our words with special caution and reflexion, and we should avoid misleading terms such as “septic arthritis”. In the example discussed here, “infectious arthritis” is a much better alternative.

## Data Availability

No datasets were generated or analysed during the current study.
